# Alfred J. Brown

**Published:** 1879-06

**Authors:** 


### OBITUARY.

*Alfred J. Brown, Sr. D. D. S.*, a Graduate of the Baltimore College of Dental Surgery, of the class of 1853, died suddenly in St. Mary's County, Maryland, May 21, 1879. Dr. Brown commenced the study of dentistry with Dr. F. H. Knapp, about the year 1841, and was one of the oldest practitioners of dentistry in Baltimore, his native city, where he had been in constant practice for a period of 36 years.

Pleasant and genial in his manners, and thoroughly devoted to the interests of his profession, Dr. Brown lived highly respected, and his death is mourned by many sincere friends.

